# A Mobile Health App (ChillTime) Promoting Emotion Regulation in Dual Disorders: Acceptability and Feasibility Pilot Study

**DOI:** 10.2196/37293

**Published:** 2023-01-27

**Authors:** Antoine Pennou, Tania Lecomte, Stéphane Potvin, Gabrielle Riopel, Camille Vézina, Marie Villeneuve, Amal Abdel-Baki, Yasser Khazaal

**Affiliations:** 1 Département de Psychologie Université de Montréal Montreal, QC Canada; 2 Centre de Recherche de l'Institut Universitaire en Santé Mentale de Montréal Montreal, QC Canada; 3 Centre Hospitalier de l’Université de Montréal Montreal, QC Canada; 4 Département de Psychiatrie Université de Montréal Montreal, QC Canada

**Keywords:** dual disorder, concomitant disorder, mental disorder, mental illness, satisfaction, mobile app, mHealth, mobile health, emotion regulation, distress management, substance use disorder, substance use, emotion, distress, mental health, acceptability, feasibility, psychosis, psychotic, schizophrenia, emotional health, pilot study

## Abstract

**Background:**

A growing number of studies highlight the importance of emotion regulation in the treatment and recovery of individuals with psychosis and concomitant disorders such as substance use disorder (SUD), for whom access to integrated dual-disorder treatments is particularly difficult. In this context, dedicated smartphone apps may be useful tools to provide immediate support to individuals in need. However, few studies to date have focused on the development and assessment of apps aimed at promoting emotional regulation for people with psychosis.

**Objective:**

The aim of this study was to evaluate the feasibility, acceptability, and potential clinical impact of a dedicated app (ChillTime) for individuals with psychotic disorders and concurrent SUD. The app design process followed recommendations for reducing cognitive effort on a mobile app. A total of 20 coping strategies regrouped in four categories (behavioral, emotional, cognitive, spiritual) were included in the app.

**Methods:**

This open pilot study followed a pre-post design. After the initial assessment, researchers asked participants to use the app as part of their treatment over a 30-day period. Feasibility was determined by the frequency of use of the app and measured using the number of *completed* strategies. Acceptability was determined by measuring ease of use, ease of learning, satisfaction, and perceived utility at the end of the 30-day study period based on responses to satisfaction questionnaires. Clinical scales measuring emotion regulation, substance use (ie, type of substance, amount taken, and frequency of use), and various psychiatric symptoms were administered at the beginning and end of the 30-day period.

**Results:**

A total of 13 participants were recruited from two first-episode psychosis clinics in Montreal, Quebec, Canada. All participants were symptomatically stable, were between 18 and 35 years of age (mostly men; 70% of the sample), and had a schizophrenia spectrum disorder with a comorbid substance use diagnosis. A total of 11 participants completed the study (attrition<20%). Approximately half of the participants used the tool at least 33% of the days (11-21 days). Cognitive and emotion-focused techniques were rated the highest in terms of usefulness and were the most frequently used. The majority of participants gave positive answers about the ease of use and the ease of learning the tool. A nonsignificant association of ChillTime use with negative symptoms and drug use was observed. No other statistically significant changes were observed.

**Conclusions:**

The ChillTime app showed good feasibility (approximately half of the participants used the tool at least 33% of the days) and acceptability among people with schizophrenia spectrum disorder and SUD. Trends suggesting a potential impact on certain clinical outcomes will need to be replicated in larger-sample studies before any conclusion can be drawn.

## Introduction

Difficulties in emotion regulation have been documented in psychosis [1-5] and its most frequent concurrent disorders such as substance use disorder (SUD) [6-9], depression [10-12], trauma-related issues [13,14], and borderline personality disorder [15,16]. Emotion regulation skills can significantly impact clinical and social recovery [17,18]. Emotion regulation difficulties may alter the management of distress associated with hallucinations [19,20] or other psychotic symptoms [21], and can be associated with increased relapse rates [22]. Distress management skills have also been associated with social functioning and cognitive difficulties such as processing speed and working memory [23,24]. Among factors that may explain these difficulties, some studies have reported that people with psychosis tend to use inefficient emotion regulation strategies [5]. A recent systematic review reported that patients with psychosis tended to more frequently use nonadaptive emotion regulation strategies such as suppression, rumination, and self-blame rather than effective strategies such as distraction and cognitive reappraisal compared to healthy controls [25]. This review also reported an association between nonadaptive emotion regulation strategies and positive symptoms. Furthermore, emotion regulation difficulties are known to be more pronounced in patients with a concurrent SUD [26], a comorbidity frequently encountered in psychosis-related disorders, which is present in approximately 50% of individuals with psychosis [27-32]. One of the explanations put forward for this phenomenon is that substances are often used as a coping strategy to self-regulate dysphoria and negative emotions [26]. Interventions aimed at improving emotional regulation skills could potentially reduce distress associated with psychotic disorders and their comorbidities.

Global access to mental health treatment is known to be problematic in many settings, even in developed countries [33]. This reality is even more pervasive among individuals with SUD [34] and psychosis [35]. In this context, individuals with concurrent psychosis and SUD face several difficulties in seeking treatment and very few settings offer integrated treatment for their comorbidities [36]. This situation has the effect that individuals with concurrent disorders, also called dual disorders, are often either not eligible for specialized treatments targeting one issue at a time or find themselves put on long wait lists in the few centers that can offer integrated treatment. Furthermore, access to interventions targeting emotional regulation interventions is even scarcer.

The development of mental health apps has grown rapidly in recent years [37]. This intervention format offers several advantages for the user (eg, obtaining services anonymously, easy and timely accessibility, no need for transportation, receiving the service at the moment it is needed), as well as for the mental health practitioner or researcher (eg, better ecological validity of the phenomena measured, elimination of memory bias, augmenting therapy by real-life training) and health system (ie, offering potentially easier access to treatment) [18]. Interest for these tools has also been observed among patients with psychosis who have expressed interest in the implementation of mental health care apps in conjunction with their usual treatment [38]. Recent reviews have illustrated that few validated mobile mental health apps were available for people with psychosis and those with commonly associated comorbidities [18,37]. Recent research on mental health apps for psychosis have focused on evaluating whether the implementation of apps is well received by patients. Studies have reported a good level of acceptability and feasibility of apps aimed at collecting clinical data in natural environments using an ecological momentary assessment approach [39,40] and tools aimed at intervening on certain specific problems such as loneliness [41] or consolidation of skills taught in therapy [42]. Other apps have also been associated with a decrease in relapse rates, hospitalization, and improved treatment adherence [43,44]. Conversely, recent data show that some mobile apps could potentially aggravate paranoid thoughts and that some individuals would be more reluctant to use this type of tool in connection with such thoughts [45,46].

Despite a significant increase in the number of mobile apps in the mental health field, only a paucity of studies have focused on the validation of apps aimed at improving emotional regulation or coping skills [47]. Furthermore, even fewer apps have been adapted specifically for individuals with cognitive deficits such as those seen in psychosis, which greatly limits the availability of tools available to this population [48]. To our knowledge, no mobile app for emotional regulation has been adapted and made available for individuals with psychosis. Knowing that individuals with psychosis and SUD face major difficulties in accessing treatment [18], we aimed to develop an app adapted to this population that would include the best adaptive emotional regulation strategies reported in the literature. Here, we first describe the steps of app development, followed by presentation of a pilot study aiming to evaluate its feasibility, acceptability, and potential clinical impacts in individuals with psychotic disorders and concurrent SUD.

The overall aim of this study was to first describe the rationale and development of a mobile app (ChillTime) aiming to provide assistance to people with a dual diagnosis of psychosis and SUD who are currently engaged in outpatient rehabilitation after a first psychosis episode. Second, this study aimed to evaluate the feasibility, acceptability, and potential impact of this smartphone app intervention aiming to promote better emotional regulation for psychological distress in individuals with psychotic disorder and concurrent SUD treated in an outpatient setting in the greater Montreal (Quebec, Canada) area.

## Methods

### ChillTime App

#### App Design and Construction

The app design process followed Rotondi et al’s [48] recommendations for reducing cognitive effort on a mobile app. It uses simple, short, and concrete language and presentations across all sections. All navigation interfaces were designed with the same presentation template to decrease confusion and facilitate navigation. The background and item color themes were all standardized, without adding distractors. All text and audio are in French. We also limited the number of pages to go through to access an exercise (two “clicks” maximum) and the number of categories per page to facilitate navigation.

Because we wanted the tool to be accessible in as many contexts as possible, it was designed to be accessible when the user would not have access to an internet connection. The last point could be of particular interest for people with low income or for people who are not within the range of a Wi-Fi connection to use the app without using their data.

Visuals (ie, app pages, alert messages), page sequences (ie, path after a “click”), video, and text for the audio exercises were created by the first author (AP) under the supervision of TL and YK. The team partnered with the *open house* studio for the recording of the audio exercises; the instructions on the reading rhythm and tone of voice were developed by AP. We then worked in close collaboration with a computer engineering firm (*psyX* innovation) to develop the app and its various functionalities (eg, creation of a patient user account, welcome email upon creation of the patient user account, reset of lost passwords by sending an email, notification system [alerts] for mood evaluation, strategy modules, strategy evaluation module, user profile module, log tables, web access to log tables by the research team). Meetings with AP and the engineering team were held to adjust the preliminary versions of the app. The legal team of the research center of l’Institut Universitaire en Santé Mentale de Montréal (IUSMM) ensured that the copyright of the product reverts to the research center that the majority of the authors are affiliated with.

#### Notification System for Mood Assessment

The system was programed to offer mood assessment via two notifications per day at different times during the week (eg, Monday 10 AM and 3 PM, Tuesday 2 PM and 6 PM). Users who had made a negative assessment of their current state (assessed on a 3-point Likert scale [ie, good, neutral, bad]) were automatically directed to the app and offered a strategy adjusted to their preferences (see Strategy Assessment section below for details on customizing the choices of strategies). The user could also decide to initiate the use of the app without first being notified.

#### Strategy Assessment

To customize the app to each person’s preferences, the artificial intelligence component memorizes the user’s previous preferred strategies. Toward this end, the user has to assess each completed strategy on a 3-point Likert scale (ie, good, neutral, bad) immediately after its use. After the user has assessed 20 strategies, the app is programed to propose the use of a strategy that has been positively evaluated on 75% or more occasions after their next mood assessment (eg, if a user reports not feeling well, the strategies they preferred in the past will be offered first, as well as a randomly selected strategy to encourage the user to try a new, less familiar strategy). To ensure confidentiality, no information about the user’s preferences is stored on the phone, which is stored on a server accessible only to the research team.

**Figure 1:**
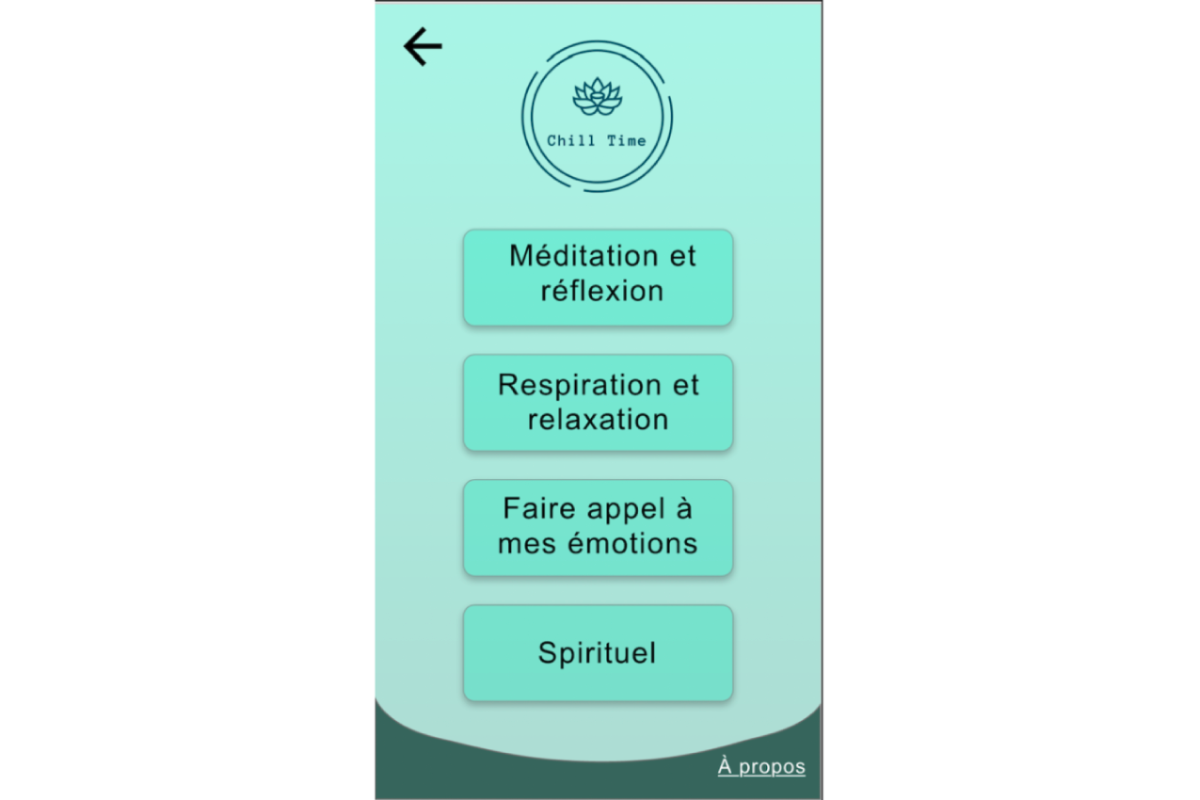
Menu of the four categories of coping strategies. From top to bottom: Meditation and reflection, Breathing and relaxation, Sharing my emotions, Spiritual.

**Figure 2:**
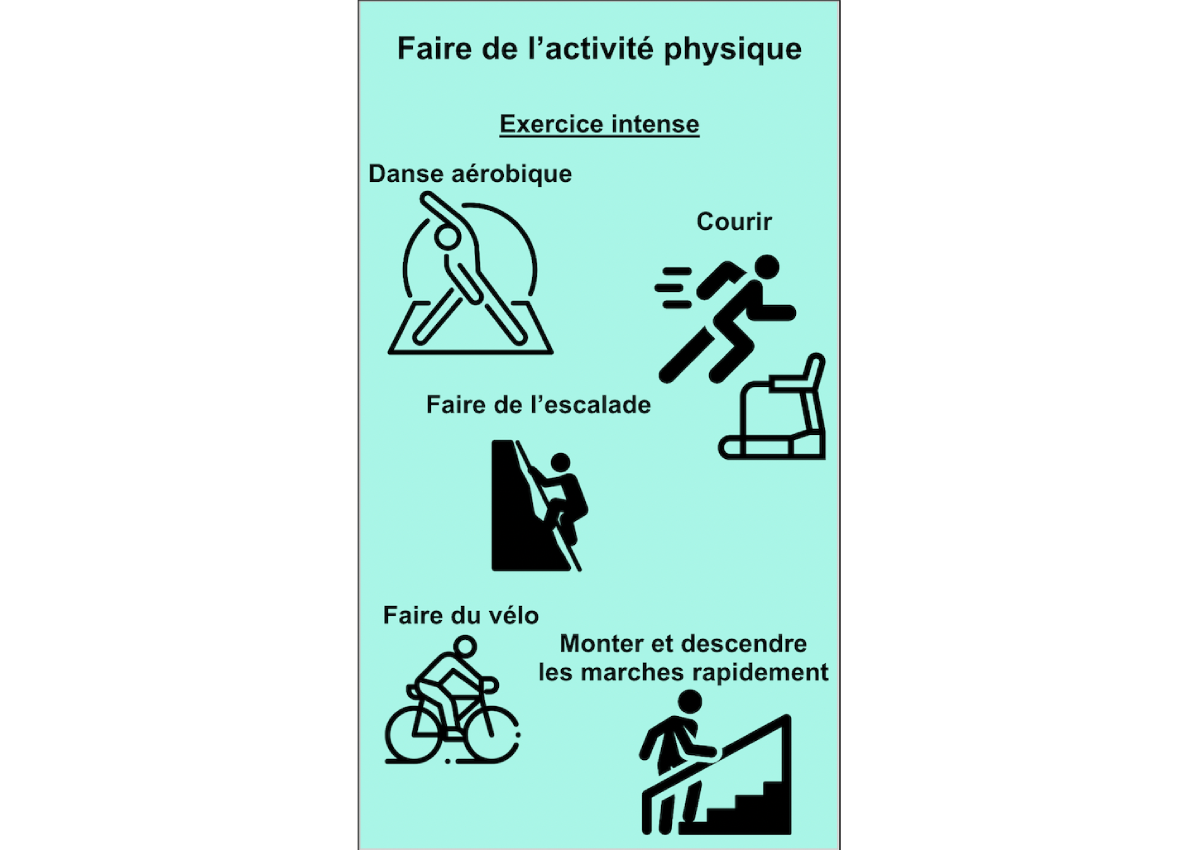
Physical exercises. From top to bottom: Intense exercise: aerobic dance, running, climbing, biking, quickly going up and down stairs.

**Figure 3:**
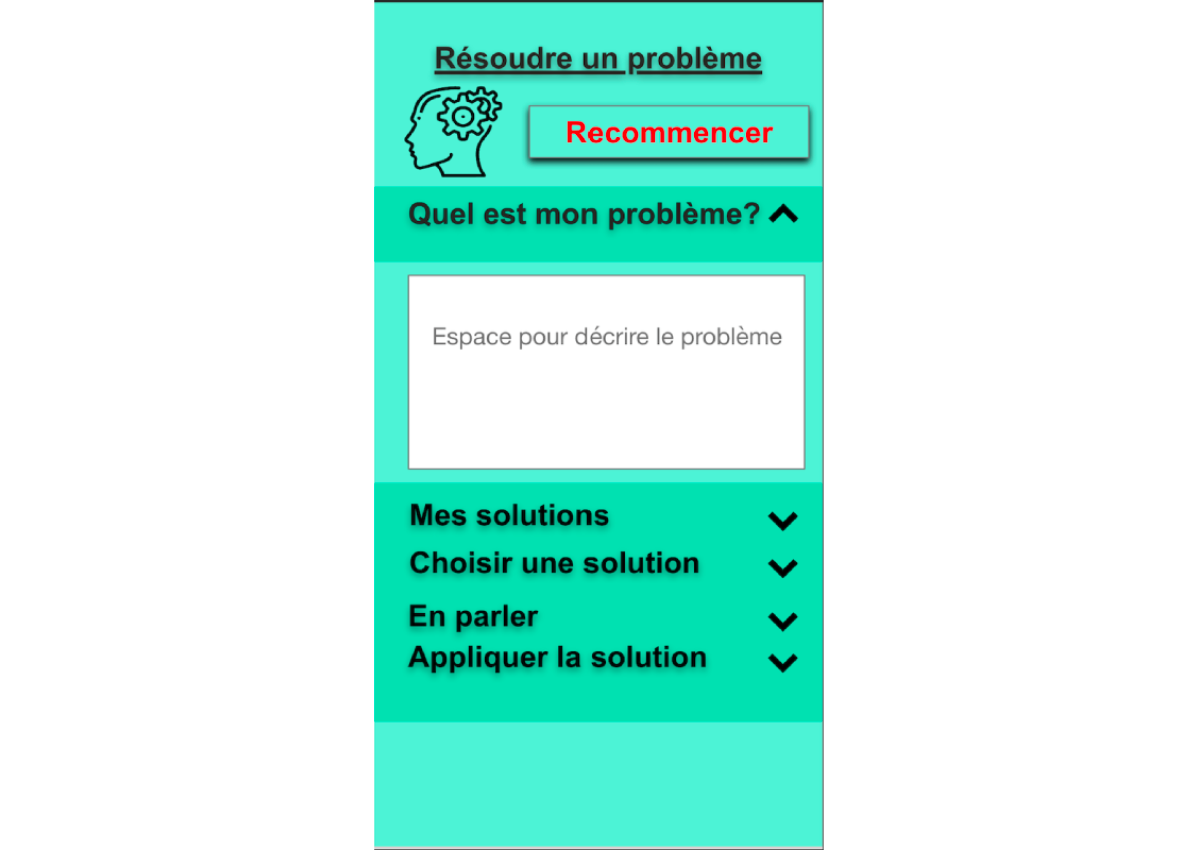
Problem solving. From top to bottom: Restart (in red), What is my problem? Space to describe the problem (white box), My solutions, Choose a solution, Talk about it, Apply the solution.

**Figure 4:**
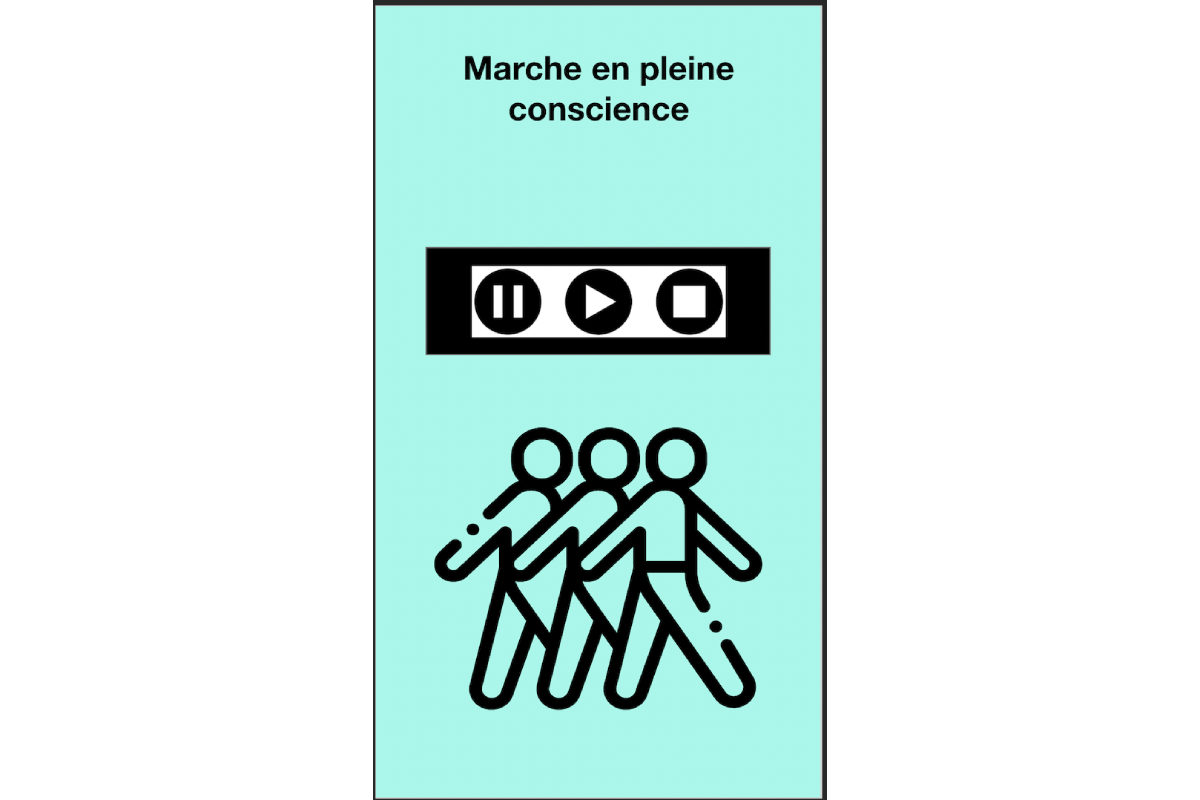
Mindful Walking.

The selection of exercises was extracted from the literature [49-57] based on the following three criteria: easy to use anywhere, anytime; does not require planning or support; and easy to understand. The choice of strategies was informed by third-wave cognitive behavioral therapy approaches and determined by the research team. We chose these approaches because of the encouraging results reported in interventions for issues related to psychosis and substance use [52,58,59]. A total of 20 coping strategies (selected by the research team among the most effective recognized strategies [49-57]) were divided into the following four categories (Figure 1): (1) *behavioral (physical)*, including diaphragmatic breathing, cardiac coherence, physical exercise (Figure 2), progressive muscle relaxation, and diving exercises; (2) *emotional*, including sharing my emotions, self-compassion, hugs, seeking laughter, and pleasant activities for the five senses; (3) *cognitive*, including problem solving (Figure 3), mindfulness strategies (body scan, self-observation, mindful walking (Figure 4), mindful breathing), and visualizing a safe place; and (4) *spiritual*, including praying, forgiveness, seeking spiritual advice from a spiritual leader, and reading religious texts. Different strategies were presented in different formats (ie, texts, videos, and audio tracks) to better adjust to the user’s preferences.

#### Beta Version of the App: Focus Group

To ensure that we considered the user experience and users’ preferences in the app design, a focus group consisting of four participants was conducted using a beta version of the app. The research team contacted the administrators of the first-episode psychosis clinic at the IUSMM to recruit patients. They were asked to present the project to various clinicians who could then refer us to the patients who had expressed an interest in participating in the focus group.

This phase consisted of a 2-hour meeting that was structured in two parts: (1) 1-hour use of the app and (2) completion of a satisfaction questionnaire inspired by the work of Ben-Zeev et al [60] to measure the usefulness and ease of use, along with an open-ended questionnaire on what they liked about the intervention (ie, ease of use, relevance of the exercises, simplicity of the instructions, difficulties they might have faced during the short practice time, best time of the day to use the app) to have the opportunity to receive input from the participants on what they would change about to the app for it to be better suited to their needs. Questionnaires were provided individually (not in a group format). The data collected indicated that the participants found the app to be easy to use and potentially useful in managing their anxiety. Participants reported bugs of the videos that were adjusted by the team before beginning the pilot study.

### Pilot Study

#### Design and Procedures

This open pilot study followed a pre-post style design. After the initial assessment, researchers asked participants to use the app as part of their treatment over a 30-day period. A research assistant contacted participants by phone during the trial period to provide support and answer questions about using the app when they did not show any activity for more than 5 days (ie, no assessments and/or exercises recorded on the app manager net interface [managed by the team] for more than 5 days).

#### Participant Recruitment

The research team first distributed advertisements to the heads of the four hospital units (ie, two outpatient clinical services and two inpatient units) involved and met with the clinical teams to present the project. Clinicians approached their patients to see if they were interested and then referred them to the researchers after they expressed interest in participating. No further involvement (eg, encouragement of app use) was requested from the clinicians.

Inclusion criteria were a dual diagnosis of a schizophrenia spectrum disorder with an SUD diagnosed by the treating psychiatrist, less than 35 years old (age limit in the participating clinics), interest in the study, and informed consent. Participants with neurological disorders, vision problems, or significant motor problems that could compromise the ability to handle a smartphone were excluded.

**Figure 5:**
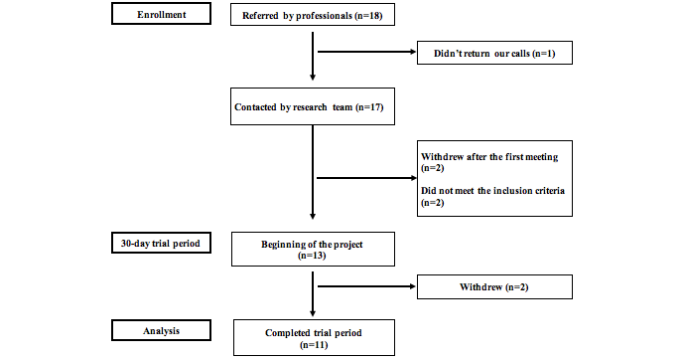
Flow of participants for the given study procedure.

The participant flow chart is shown in Figure 5. A total of 18 patients were referred to us by professionals at participating centers (convenience sample). The research team was able to contact 17 potential participants; one participant did not return our calls. Two participants withdrew after the first meeting (ie, focus group on the beta version) with the research team stating that they were no longer interested in participating, and two other participants were excluded because they did not meet the inclusion criteria established for this study. Of the 13 participants included at the beginning of the pilot study (ie, “beginning of the project” in Figure 5), two withdrew after the first meeting. One of them mentioned that he wanted to end his participation because the project “wasn’t his kind of thing” and the other patient did not give a reason for withdrawal. Analyses were performed on the remaining 11 study completers.

#### Participant Characteristics

Information regarding the age, gender, marital status, education level, vision disorders, and other basic characteristics was collected using a sociodemographic questionnaire. The treating psychiatrist diagnostic was confirmed by a doctoral student in psychology using the Structured Clinical Interview for Diagnostic and Statistical Manual of Mental Disorders-5 (SCID-5) manual for diagnosis.

A total of 13 individuals were recruited from two first-episode psychosis clinics (inpatient and outpatient) in Montreal (ie, Clinique Connect TI–IUSMM, Clinique JAP–Centre Hospitalier de l’Université de Montréal [CHUM]). Participants’ ongoing treatment consisted of a 3-year follow-up in which patients are supervised by a team composed of psychiatrists, occupational therapists, psycho-educators, and social workers. The objectives of this program are: symptomatic stabilization, integration into work and studies, education about psychosis, and education for the patients’ families. All participants included in the study were symptomatically stable. Participants were between 18 and 35 years of age and had a schizophrenia spectrum disorder with a substance use diagnosis (Table 1). Two participants mentioned having no drug use problems during the SCID interview (we indicated the diagnosis of the treating psychiatrist), but acknowledged substance use. Cannabis and alcohol were the most commonly used substances. Among the 11 participants, 5 (46%) participants reported using cannabis daily or almost daily. Table 1 shows more details on the types of the most commonly used substances within the last 12 months and the other clinical and demographic characteristics.

**Table 1:** Participant characteristics (N=11).

Characteristic		Value
Age (years), mean (SD)		27.6 (4.5)
Gender (men), n (%)		9 (82)
Education (years), mean (SD)		9.7 (2.0)
Marital status (single), n (%)		10 (91)
**Diagnosis, n (%)**		
	Schizophrenia only	1 (9)
	Schizophrenia+SUD^a^	6 (55)
	Schizophrenia+SUD+MDD^b^	2 (18)
	Schizoaffective disorder+SUD	1 (3)
	Psychosis NOS^c^	1 (9)
**ASSIST^d^: substances taken at least 1 or 2 times in the past 3 months, n (%)**		
	Alcohol	9 (82)
	Cannabis	10 (91)
	Amphetamine	5 (46)
	Cocaine	4 (36)
	Painkillers or sleeping pills	2 (18)
	Hallucinogens	2 (18)

^a^SUD: substance use disorder.

^b^MDD: major depressive disorder.

^c^NOS: schizophrenia spectrum and other psychotic disorder not otherwise specified.

^d^ASSIST: Alcohol, Smoking and Substance Involvement Screening Test.

### Data Collection

The analysis consisted of between-subject comparisons in a pre-post prospective design. No masking was used to blind the interviewers to the study variables. Data collection (ie, semistructured interviews and questionnaires) was carried out by three authors (AP, GR, and CV). AP was also responsible for the initial contact with the participants and for providing technical support during the trial period. Data were collected by face-to-face meetings, teleconference, or by telephone (“pre” time period). All participants were informed of the study goals and signed a consent form at the first interview. The meetings lasted between 2 and 2.5 hours. The recruitment time frame was from January to November 2020. The scales measuring symptomatic presentations (ie, SCID-5, Brief Psychiatric Rating Scale-Expanded [BPRS-E]) were administered by a trained interviewer. The interviewer introduced the mobile app and provided information on its functioning after administering all scales. The second meeting (ie, “post” time period) followed the same format with the addition of the acceptability measures presented below.

### Measures

#### Feasibility

Feasibility was determined by the frequency of use of the app and was measured using the number of *completed* strategies (eg, listening to the entire length of a meditation recording and clicking a button to confirm that the exercise was completed). Moments of interaction with the app (eg, opening the app and looking at different exercises without selecting any of them) or partially completed exercises (eg, listening to only half of the meditation recording) were not counted as moments of use. We also included a measure of attrition as an index of feasibility. Based on previous studies of cannabis users [61] and cannabis users who have recently experienced a psychotic episode [62], we estimated that attrition would reach a maximum of approximately 30%.

#### Acceptability

Acceptability was determined by measuring ease of use, ease of learning, satisfaction, and perceived utility at the end of the 30-day study period by using the same scales as used in the focus group: (1) a satisfaction questionnaire inspired by the work of Ben-Zeev et al [60] and (2) a questionnaire with open-ended questions assessing participant impressions of what they liked about the intervention and what they would change.

#### Clinical Scales

The clinical scales were administered at the beginning and end of the 30-day study period with the exception of the Alcohol, Smoking and Substance Involvement Screening Test (ASSIST), which was administered only at the beginning of the trial period.

The Cognitive Emotion Regulation Questionnaire (CERQ) was used to assess nine cognitive dimensions of emotional regulation (self-blame, acceptance, rumination, positive centering, action centering, positive reappraisal, perspective taking, dramatization, blame others). This instrument consists of 36 items rated on a 5-point Likert-type scale ranging from “almost never” to “almost all the time” (internal consistency: α=.75-.87 depending on the subscale) [63]. The questionnaire also included second-order factors grouped into adaptive (ie, acceptance, positive refocusing, refocus on planning, positive reappraisal, and putting into perspective) and less-adaptive (ie, self-blame, rumination, catastrophizing, and blaming others) strategies (internal consistency: α=.91 for adaptive and α=.87 for less-adaptive strategies [61]).

The BPRS-E [62] was used to measure different psychiatric symptoms (eg, positive, negative, anxiety, depressive symptoms). The instrument is composed of 24 items rated on a 7-point Likert-type scale ranging from “not present” to “extremely severe” (internal consistency: α =.81 for positive symptoms and α=.91 for negative symptoms).

The semistructured Time-Line Follow Back (TLFB) interview [64] was used to assess the age of onset of use, and the amount and category of substances the participant may have used in the last month for each week. In terms of metrological qualities, correlations as high as 0.96 in validity and test-retest reliability have been reported for this instrument [65].

The ASSIST [66] was used to assess the severity of addictions. This instrument consists of eight questions addressing tobacco, cannabis, cocaine, stimulants, hallucinogens, opiates, and other drug use. The dimensions assessed through these eight questions are: (1) the substance used, (2) the frequency of use, (3) the desire to use (“craving”), (4) psychosocial problems that could have resulted from use, (5) difficulties in fulfilling one’s obligations related to use, and (6) other problems that could be related to drug use. Questions related to each theme are rated on a 5-, 3-, or 2-point Likert-type scale (internal consistency of the French version: α=.93) [67].

### Statistical Analysis

Descriptive statistics are used to report the frequency and distribution of the different patient characteristics (diagnoses, sociodemographic variables, and clinical variables), as well as the frequency of use and perceived usefulness. The difference between the measurements at the beginning and end of the 30-day study period on the clinical and emotion regulation scales was assessed using a *t* test for paired data.

### Ethics Considerations

The study was approved by the respective ethics committees of the research centers of the IUSMM (approval number MP-12-2018-1420) and the CHUM (approval number MEO-12-2021-9143). All participants signed a consent form.

## Results

### Feasibility

Participants used (ie, frequency of use of the app and the number of *completed* strategies) the app on average 33% of days during the project period. Approximately half of the patients (5/11) used the tool at least 33% of the days (11-21 days) while the other half used it 27% of the days or less (5-8 days) during the project. Data from two participants could only be partially recorded because of difficulties in logging into the app’s user account. The attrition rate was 18%.

### Acceptability

The frequency of use of the different categories of exercises and their evaluation by users are presented in Table 2. In terms of preferences of exercise categories, cognitive and emotion-focused techniques were rated the highest in terms of usefulness and were the most frequently used. The behavioral exercises were used slightly less frequently. The “spiritual” category was the least used and also received the lowest score. The majority of participants gave positive answers about the ease of use (eg, 81.8% agreed with the statement “Overall, using ChillTime was easy”) and the ease of learning the tool (eg, 81.8% agreed with the statement “I feel it is easy to learn to use ChillTime”). Furthermore, 90% of participants would recommend ChillTime to a friend. In terms of satisfaction, 55% responded that ChillTime is a tool that they would use daily and 73% indicated that the different sections of ChillTime complemented each other well. In terms of usefulness, 55% indicated that ChillTime had helped them in difficult times.

In their responses to the open-ended acceptability questionnaire on their impressions of the app at the end of the 30-day trial period, several participants mentioned the beneficial effect of the app in calming their anxiety. More specifically, the mindfulness meditation and cardiac coherence exercises were often named as being particularly useful for this purpose. Some respondents also specified that they would rather use the app when needed (eg, during a crisis) than on a daily basis. In terms of exercise format, several participants mentioned that they particularly appreciated the exercises with audio or simple animation. In terms of difficulties encountered in using the app, many participants pointed out that it was difficult for them to carry out the exercises outside their house because of distractions and ambient noise (eg, noise in the subway).

**Table 2:** Relative overall use and subjective utility of the different categories of exercises used.

Category	Usage (%)	Average rating^a^
Cognitive	36	2.54
Behavioral	21	2.42
Emotion-focused	34	2.67
Spiritual	9	2.20

^a^Subjective usefulness assessed on the app with a 3-point Likert scale (1=not useful, 2=neutral, 3=useful).

### Potential Clinical Effects

The results on the clinical scales are presented in Table 3. A nonsignificant reduction was found in the pre-post level of the negative symptoms scale of the BPRS-E in the paired sample *t* test (*P*=.05). No significant difference between pre and post scores on the CERQ and the TLFB were found.

**Table 3:** Questionnaire scores and clinical variables at baseline (T0) and after the 30-day trial period (T1).

Variable		T0, mean (SD)	T1, mean (SD)
Brief Psychiatric Rating Scale-Expanded (BPRS-E): negative symptoms		7 (2.07)	5.9 (1.46)
**Cognitive Emotion Regulation Questionnaire (CERQ)**			
	Use of more adaptive strategies	69.25 (14.73)	65.69 (15.46)
	Use of less adaptive strategies	39.19 (6.04)	38.0 (7.84)
**Time-Line Follow Back (TLFB): number of days used (30-day period)**			
	Cannabis	17.29 (11.47)	10.25 (9.88)
	Amphetamine	18.33 (13.43)	6.00 (12.33)
**TLFB: total amount used (30-day period)**			
	Cannabis (grams)	23.29 (25.43)	17.00 (25.59)
	Amphetamine (number of pills)	26.00 (34.38)	3.00 (6.16)

## Discussion

### Principal Findings

#### Design

This study aimed to evaluate the feasibility, acceptability, and potential impact of the ChillTime app intervention to promote better emotional regulation for psychological distress in individuals with psychotic and concurrent substance use disorders treated in an outpatient setting in the greater Montreal area. The app was designed on the base of Rotondi et al’s [48] recommendations for reducing cognitive effort on a mobile app.

#### Feasibility

Our results suggest that the use of ChillTime appears feasible. The frequency of use of the app was lower than that found in other studies assessing apps targeting social anxiety [41] or psychosis [60]. This difference could be explained in part by a more stringent method of measurement used to record usage. In this study, only completed exercises were compiled by the app, which differs from studies that count every time participants interact with the tool (eg, [60]). In addition, the fact that several participants reported in the open-ended acceptability questionnaire that ChillTime requires (for some exercises) a quieter environment could also have contributed to lower usage than a tool that primarily employs self-monitoring [40]. Indeed, completing a self-reported questionnaire is less likely to require an environment free of ambient noise than listening to an audio track of guided meditation. These results suggest that the feasibility of ChillTime could potentially be improved if more exercises were adapted to environments with ambient noise (eg, more text than audio recordings). Alternatively, the app could subdivide the exercises according to the environment (ie, “exercises to be done in a quiet place” and “exercises for noisier places”). It is also possible that a learning effect influenced the frequency of use of the app (ie, a participant having memorized the technique does not feel the need to use the app as much). In our small sample, half of the participants used ChillTime more than 33% of the days throughout the study. These results are consistent with results reported in similar studies with cannabis users [68] and cannabis users who had a recent psychotic episode [69]. These results are also consistent with the expected completion rate in a population with dual disorder (with cannabis use) [70]. A larger study may allow us to replicate this usage pattern and see whether it is associated with certain patient characteristics. This could enable better targeting toward individuals who are more likely to benefit from the app. Attrition was within the acceptable range.

#### Acceptability

Our results suggest that ChillTime is acceptable to participants. These results are consistent with other studies that have used mobile apps with a similar population [39-41,60]. The preference in terms of categories may be partly explained by the fact that the three most popular categories (ie, behavioral, emotional, and cognitive) contained different strategy formats, including an audio format (which was appreciated by the participants). This contrasts with strategies in the spiritual category that were only in text format.

It is important to mention that such preferences need to be replicated before drawing generalizable conclusions, as such preferences are possibly influenced by cultural aspects. Nevertheless, this study is one of the few that assessed user preferences for different emotion regulation strategies. Most of the previous apps-related intervention studies investigated some strategies but for a specific problem (eg, medication adherence [60], loneliness [41]) or a single bundle of strategies (eg, dialectical behavior therapy [47]). Once our results are replicated, these insights could contribute toward optimizing the app for this population.

The ChillTime app was frequently used by a portion of the participants and, based on the responses on the qualitative questionnaire, was considered useful for managing certain symptomatic manifestations (ie, anxiety). It was also judged as a tool with good acceptability.

#### Potential Clinical Effects

Although the differences on the clinical scales did not reach the significance threshold, trends were observed. Among them, we can question the potential effect of the app on the reduction of negative symptoms. A future study with a larger sample would allow us to confirm this trend. Furthermore, a reduction in negative symptoms has also been observed in other studies using an app tested among individuals with psychosis (ie, [60]). To our surprise, no significant differences were observed on the emotional regulation abilities assessed by the CERQ. It is possible that the short duration of the study (ie, 30 days) was not sufficient to promote change in the various skills assessed or that the emotion regulation measure was not close enough to the strategies used in the app. In terms of substance use, measured by the TLFB, the differences observed between the two measurement periods did not produce significant differences. This could be partly due to the high variability of the different observations and the small sample size. Although not significant, the large difference in terms of quantities and frequency of drug use points to a potential effect of the app on drug use, which would also need to be confirmed in a larger study.

### Limitations

This study has several limitations when compared to a true outcome study. The sample size of the study was small and there was no randomization or control group. These limitations make it difficult to interpret the results on the clinical scales (eg, to determine the representativeness of the sample). A larger study is needed to replicate the results and determine the potential efficacy or real-world effectiveness of the app in helping people with comorbid psychotic and substance misuse disorders improve in emotion regulation. In addition, the study was conducted over a short period of time (ie, 30 days). Some processes (eg, affective regulation capacity) may take longer to change in a given individual, therefore requiring longer studies. Another limitation of the study is the recruitment strategy. Since this was a convenience sample, only those interested in participating were recruited. Therefore, this sample may not be representative of the population. It would also be important for a future study to further explore the characteristics of participants who used the app less to better understand why their level of participation was lower.

### Conclusion

Despite these limitations, this study shows encouraging results for the relevance of pursuing the development and studies of apps that provide immediate support to patients with psychosis and concomitant substance use disorders presenting emotional regulation difficulties.

## Abbreviations

ASSIST: Alcohol, Smoking and Substance Involvement Screening Test
BPRS-E: Brief Psychiatric Rating Scale-Expanded
CERQ: Cognitive Emotion Regulation Questionnaire
CHUM: Centre Hospitalier de l’Université de Montréal
IUSMM: l’Institut Universitaire en Santé Mentale de Montréal
SCID-5: Structured Clinical Interview for Diagnostic and Statistical Manual of Mental Disorders-5
SUD: substance use disorder
TLFB: Time-Line Follow Back
